# Iron-Related Genes and Proteins in Mesenchymal Stem Cell Detection and Therapy

**DOI:** 10.1007/s12015-023-10569-3

**Published:** 2023-06-03

**Authors:** Kosha J. Mehta

**Affiliations:** Centre for Education, Faculty of Life Sciences and Medicine, King’s College London, London, UK

**Keywords:** Mesenchymal stem cells, Iron, Reporter genes, Ferritin, Transferrin receptor, Lipocalin-2, Hepcidin

## Abstract

Mesenchymal stem cells (MSCs) are located in various tissues of the body. These cells exhibit regenerative and reparative properties, which makes them highly valuable for cell-based therapy. Despite this, majority of MSC-related studies remain to be translated for regular clinical use. This is partly because there are methodical challenges in pre-administration MSC labelling, post-administration detection and tracking of cells, and in retention of maximal therapeutic potential in-vivo. This calls for exploration of alternative or adjunctive approaches that would enable better detection of transplanted MSCs via non-invasive methods and enhance MSC therapeutic potential in-vivo. Interestingly, these attributes have been demonstrated by some iron-related genes and proteins.

Accordingly, this unique forward-looking article integrates the apparently distinct fields of iron metabolism and MSC biology, and reviews the utility of iron-related genes and iron-related proteins in facilitating MSC detection and therapy, respectively. Effects of genetic overexpression of the iron-related proteins ferritin, transferrin receptor-1 and MagA in MSCs and their utilisation as reporter genes for improving MSC detection in-vivo are critically evaluated. In addition, the beneficial effects of the iron chelator deferoxamine and the iron-related proteins haem oxygenase-1, lipocalin-2, lactoferrin, bone morphogenetic protein-2 and hepcidin in enhancing MSC therapeutics are highlighted with the consequent intracellular alterations in MSCs. This review aims to inform both regenerative and translational medicine. It can aid in formulating better methodical approaches that will improve, complement, or provide alternatives to the current pre-transplantation MSC labelling procedures, and enhance MSC detection or augment the post-transplantation MSC therapeutic potential.

**Graphical Abstract:**

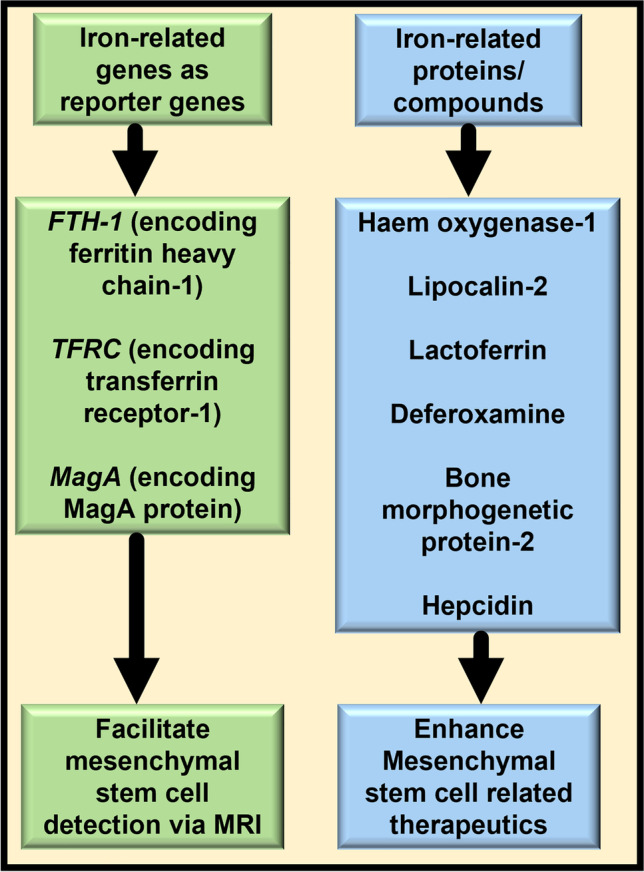

## Introduction

Iron in essential for various intracellular activities, and stem cells would be no exception. For example, ribonucleotide reductase is an enzyme that facilitates DNA synthesis and repair, and iron is a cofactor for this enzyme. Also, iron is essential for mitochondrial respiration. It is used in the synthesis of haem and [Fe-S] clusters; specifically, cytochrome c that not only participates in the electron transport chain, but also has a role in apoptosis [1]. Cytochrome P450 are a group of enzymes that utilise haem (iron) as a cofactor, and these enzymes play an important role in detoxification/metabolism of drugs [2]. The enzyme catalase possesses haem groups (containing iron) and this enzyme is an important anti-oxidant as it converts hydrogen peroxide to water and oxygen, and thereby prevents/reduces cell damage by free radicals [3]. Amongst specific examples of the involvement of iron at cellular level include the incorporation of iron within haemoglobin in maturing erythrocytes, and thereby aiding in oxygen transport throughout the body. Iron is also a part of myoglobin found in skeletal and cardiac muscle tissue.

In a pathological context, specifically pertaining to stem cells, iron has been found to maintain cancer stem cells [4] and iron loading has been found to inhibit self-renewal of human pluripotent stem cells [5]. Also, iron and iron-related proteins play a role in Mesenchymal stem cell (MSC) biology. This includes the role of iron loading on cellular components, processes and signalling pathways of the MSCs [6].

MSCs are the most widely researched stem cell types because of their ability to support several physiological processes in the body and their exuberant reparative and regenerative properties. Located in various body tissues, these cells not only show multilineage differentiation but also secrete immune and trophic factors that stimulate endogenous repair mechanisms at the target site. Furthermore, MSCs show tropism towards tumour and inflammation/injury [6]. Expectedly, these cells have shown promising results in several in-vitro, pre-clinical and clinical trials for a wide range of pathologies including COVID-19 [7–16]

Despite their therapeutic potential, MSCs are not frequently used in clinical settings for amelioration of pathological conditions. Amongst the many reasons for this are the challenges encountered in pre-transplantation MSC labelling and post-transplantation MSC detection via non-invasive methods like the Magnetic Resonance Imaging (MRI). Nanoparticles including iron oxide nanoparticles have been used to enhance MSC detection, but their usage is confounded by various challenges [17]. Evidently, these processes involve multistep and complex approaches that are yet to be perfected.

Therefore, it is extremely important to search for effective and non-toxic approaches that not only preserve MSC functionality during extraction and in-vitro cultivation stages but also permit the detection of transplanted MSCs non-invasively and help retain and/or enhance their reparative and regenerative potential in-vivo.

Iron-related genes and proteins have shown the potential to support many of these pre-requisites for a successful MSC therapy. Thus, this review compiles and critically evaluates the usage of the iron-related genes (genes of ferritin, transferrin receptor-1 and MagA) as reporter genes because their encoded proteins allow cellular iron accumulation that eases in-vivo MSC detection and tracking via MRI. The review also addresses the roles of deferoxamine (iron chelator) and the iron-related proteins haem oxygenase-1, lipocalin-2, lactoferrin, bone morphogenetic protein-2 (BMP-2) and hepcidin in preserving MSC characteristics in-vitro and in-vivo, and in enhancing MSC therapeutics.

## Iron-Related Genes as Reporter Genes for MSC Detection and Tracking

### Background

It is essential to be able to detect the transplanted cells and track their destination in-vivo to check the efficacy of MSC therapy. Hence, prior to transplantation, MSCs are labelled so that post-transplantation detection and in-vivo cell tracking via non-invasive methods like MRI become feasible. Cell labelling is essential as it distinguishes between the host and transplanted cells. Thus, MRI contrast agents such as iron oxide nanoparticles (IONPs) are used for cell labelling. Upon exposure to a magnetic field, IONP-labelled cells appear as darker regions in the field, allowing easy identification of areas of interest [18]. Despite the promising results shown by IONP-labelled MSCs [17, 19, 20] their usage is confounded by challenges. For e.g., although IONPs are non-toxic to MSCs and do not present other side-effects [21], long-term effects of these on MSC functionality are unknown. Moreover, with time, there may be a decrease in the MRI signal from the internalised IONPs because of cell proliferation and/or exocytosis of IONPs from the labelled transplanted cells [22]. This leads to progressive loss of the detection signal and misleads the interpretation of long-term MR images. Also, the transplanted cells may be engulfed by macrophages resulting in false positives and this approach does not detect cell differentiation. Collectively, this makes long-term tracing difficult and does not reflect the true number of transplanted cells [17, 23].

**Fig. 1:**
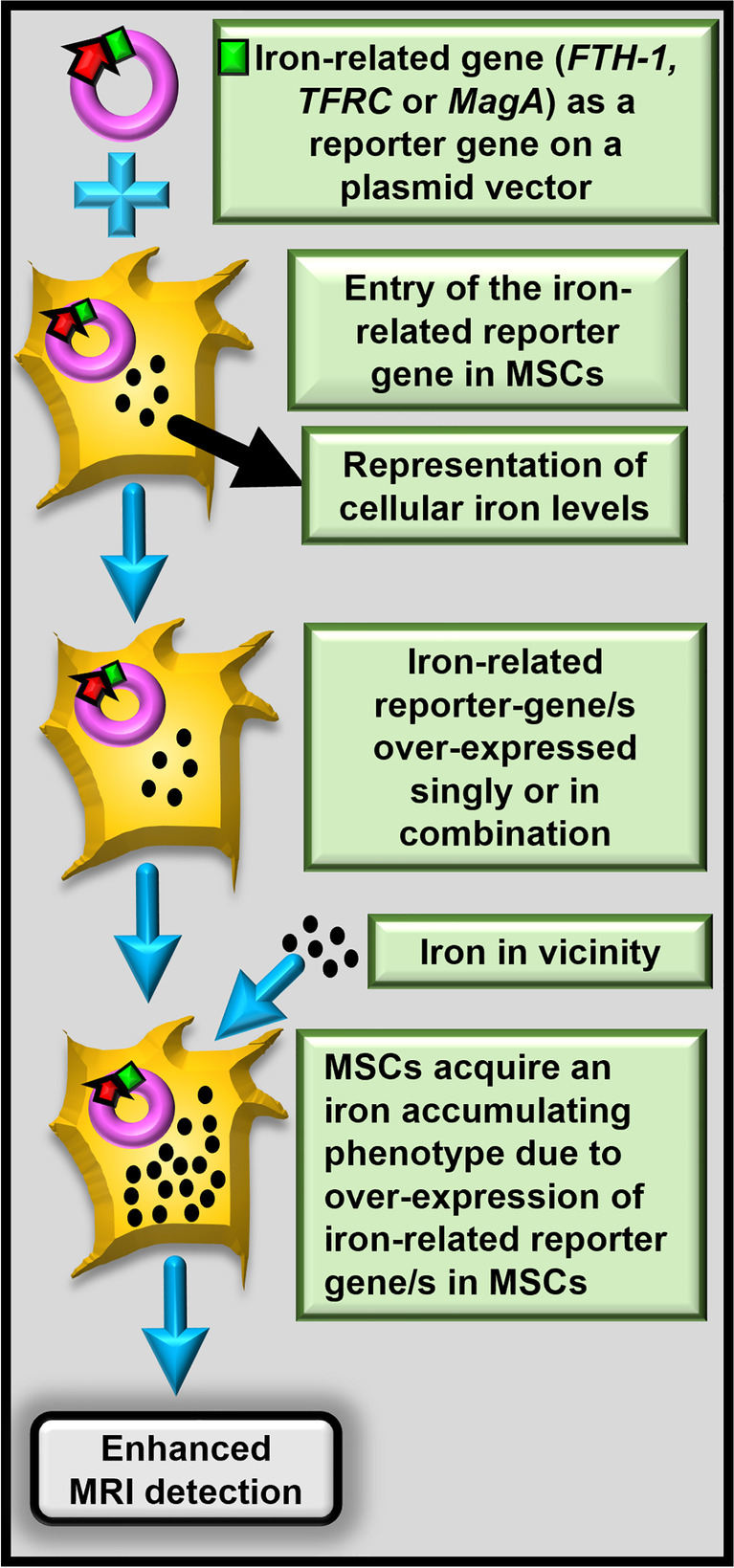
Principle of using iron-related reporter genes for MSC detection and tracking. The figure encapsulates the principle underlining the utilisation of iron-related genes (*FTH-1, TFRC* or *MagA*) as reporter genes in the MSCs to increase cellular iron accumulation and thereby improve cell detection via MRI

These constraints can be overcome by introducing and overexpressing an iron-accruing reporter gene in the MSCs prior to the MRI assessment. The term ‘reporter genes’ normally denotes reporting of a biological activity such as a signalling pathway or transcription factor binding. Here, in the context of enhancing MRI assessment following post-MSC transplantation, the reporter gene overexpressed in the MSCs imparts iron accumulation ability to the MSCs, thereby increasing the MRI signals given-off by the cell and making long-term tracing possible (Fig. 1). In this approach, signal intensity is maintained even after cell division and it can disappear after cell death, allowing for the detection of only viable cells; MRI alone cannot differentiate between live and dead cells. Moreover, if cell‐type‐specific promoters are introduced in the cells during cloning and overexpression, then the reporter gene can also help determine cellular differentiation status [24].

### Ferritin and Transferrin Receptor-1 Genes as Reporter Genes in MSCs

In addition to reporter-genes like tyrosinase and *β*-galactosidase, genes of iron-related proteins such as ferritin and transferrin receptor-1 (TfR-1) have been explored as reporter genes for monitoring the location and viability of transplanted MSCs via MRI. Ferritin is an iron storage protein found intracellularly and in the circulation, whereas TfR-1 is the cell surface iron importer protein, which allows regulated entry of transferrin-bound iron into the cells. Thus, the iron accrual ability of both these proteins promotes their utilisation in MSC detection via MRI.

In context of using these as reporter genes, ferritin has relatively more applications. This is partly because it can be integrated into the cell genome and its expression remains unaffected by cell division and proliferation, although integration is more dependent on the method used rather than the gene itself (e.g., transduction via lentivirus versus adeno-associated virus). Also, ferritin has two subunits; light chain and heavy chain, where the heavy chain shows stronger ferroxidase activity and can promote cellular iron uptake from the vicinity [25]. When ferritin heavy chain was used as a reporter gene in human MSCs, it allowed cellular iron accumulation and successful detection by MRI in-vivo (brain tissue). In addition, the MSCs retained surface markers, expression of self-renewal genes and multilineage differentiation ability [26]. Also, rat MSCs carrying ferritin heavy chain-1 could successfully differentiate into neuron-like cells in-vitro and increase signal intensity for MRI without altering cell viability and differentiation rate [25]. This presented ferritin heavy chain-1 as a potential reporter gene to examine neural differentiation of MSCs and promised its diagnostic application in neurological diseases. Accordingly, aiming to treat stroke, ferritin heavy chain-1 gene was transduced in rat bone marrow-derived MSCs (BM-MSCs) and injected in the internal jugular vein of rats in the direction of cranium. While the MRI signal intensity of IONP-loaded BM-MSCs (used as control) faded with time, the signal intensity in case of the ferritin-heavy-chain-1-loaded BM-MSCs was retained between 10 and 60 days. It was concluded that for long-term tracking of cells, ferritin labelling was more stable than IONP labelling [27].

However, another set of studies by Pereira et al. reported slightly different results. At physiological concentration of extracellular iron, while the overexpression of TfR-1 was well tolerated by mouse MSCs, overexpression of ferritin heavy chain-1 affected cellular iron homeostasis, reduced cell proliferation, altered cell phenotype and upregulated the endogenous TfR-1. Unexpectedly, sole overexpression of neither reporter genes (TfR-1 or ferritin heavy chain-1) led to substantial increment in intracellular iron content. Instead, supplementation of the culture medium with iron sources proved to be better at obtaining the required MRI contrast than using these reporter genes [28]. Therefore, it was concluded that ferritin heavy chain-1 may not be suitable for tracking cells in those tissues where the iron content is high enough to maintain cell viability.

Since the inability of TfR-1 as a reporter gene was attributed to its insufficient expression levels, Pereira et al. re-evaluated the potential of TfR-1 to function as a reporter gene by inducing mouse TfR-1 at high levels in Chinese hamster ovary cells that have the ability to express high levels of recombinant proteins. Major increments were observed in TfR-1 (total) and ferritin heavy chain‐1, and the intracellular iron content increased significantly, even in the absence of major iron supplementation to the culture medium. Following this, injecting the reporter-gene-labelled cells in chick embryos in-ovo showed that the MR contrast obtained by supplementing the culture medium of the control cells (without reporter gene) with ferric citrate was almost the same as that in cells with the reporter gene*.* It was thereby concluded that for short-term tracking of cells, loading the cells with ferric citrate was more effective than TfR-1 overexpression [24].

On the other hand, co-expression of these iron-related reporter genes enhanced MRI detection and retained biological properties of the transplanted MSCs. When human MSCs expressing ferritin, transferrin receptor and Deltex-1 were transplanted into rabbits with closed penile fracture, the co-expression of ferritin and transferrin receptor increased the iron accumulation capacity and provided sufficient MRI contrast for detecting the distribution and migration of MSCs. Additionally, as Deltex-1 promoted MSC differentiation into smooth muscle cells, the fracture showed healing [29]. This approach facilitated both detection and therapeutics.

Collectively, data suggest that although for short-term tracing, loading cells with a suitable iron source may be an option, for long-term longitudinal tracing, co-expression of these reporter genes may give better clinical outcomes. But this may depend on the iron content of the tissue under investigation.

### MagA as a Reporter Gene in MSCs

*Magnetospirillum magnetotacticum* is a magnetotactic bacterium, which produces single-magnetic domain crystals and incorporates these into magnetosomes [22]. MagA is an iron-regulated protein (from *Magnetospirillum magnetotacticum*), usually containing a lipid bilayer around magnetite (Fe_3_O_4_). A previous study showed that MagA is an iron transport protein involved in the synthesis of magnetic particles, and also that MagA expressing *Escherichia coli* cells accumulated iron in vesicles [30]. This was challenged by a subsequent study which showed that MagA of Magnetospirilla is not involved in the formation of magnetosomes [31]. Regardless, MagA expressing mammalian cells showed iron (Fe^2^) accumulation in magnetosome-like particles, and redistribution and aggregation of existing cellular iron, which increased the size of the magnetic particles and improved MRI sensitivity [32, 33]. Researchers have attempted to harness this property to enhance MSC detection via MRI.

Although earlier, Pereira et al. showed that the expression of *MagA* induced toxic effects in murine MSCs [34], Shen et al. showed that *MagA* gene had a huge potential as a magnetic reporter gene for MSC tracking with MRI and for improving MRI detection in-vivo [35]. In their study, in-vitro data showed that in the presence of an iron supplement, *MagA*-expressing MSCs accrued iron in vesicles and increased MRI sensitivity. Likewise, the liver of iron-loaded *MagA*-expressing transgenic mice showed high iron concentration and increased MRI sensitivity. Excess iron increases ROS and this can induce p38-MAPK signalling [36]. *MagA-*expressing MSCs showed lower increments in ferritin and p-p38 MAPK expression compared to control MSCs, and decreased the excess-iron-induced inhibition of osteogenic differentiation [35]. Thus, these results demonstrated attenuation of ROS-induced negative effects under iron-loaded conditions due to *MagA-*expression in MSCs. This opens new therapeutic avenues for treating the myriad of pathological conditions that show excessive iron loading [37].

Also, while iron-treatment inhibited MSC proliferation and osteogenic differentiation, *MagA*-expressing MSCs reduced these effects. Thus, MagA suppressed the exogenous iron-induced oxidative stress and showed the potential to attenuate iron-overload-induced injury to the bone-marrow haematopoietic microenvironment [35]. Based on this, a therapeutic approach involving *MagA* could be developed to tackle transfusion-induced iron loading. Along the same line, aiming to enhance detection by MRI and as an alternative to IONPs, kerans et al. described the transfection of MSCs with magnetosome-associated genes (*mms6* or *mmsF*) derived from *Magnetospirillum magneticum* AMB-1. These genes helped the assimilation of intracytoplasmic magnetic nanoparticles that not only facilitated MRI detection but also retained inherent MSC proliferation, differentiation and migration [22].

## Iron-Related Proteins and Compounds Enhance MSC Therapeutics

**Fig. 2:**
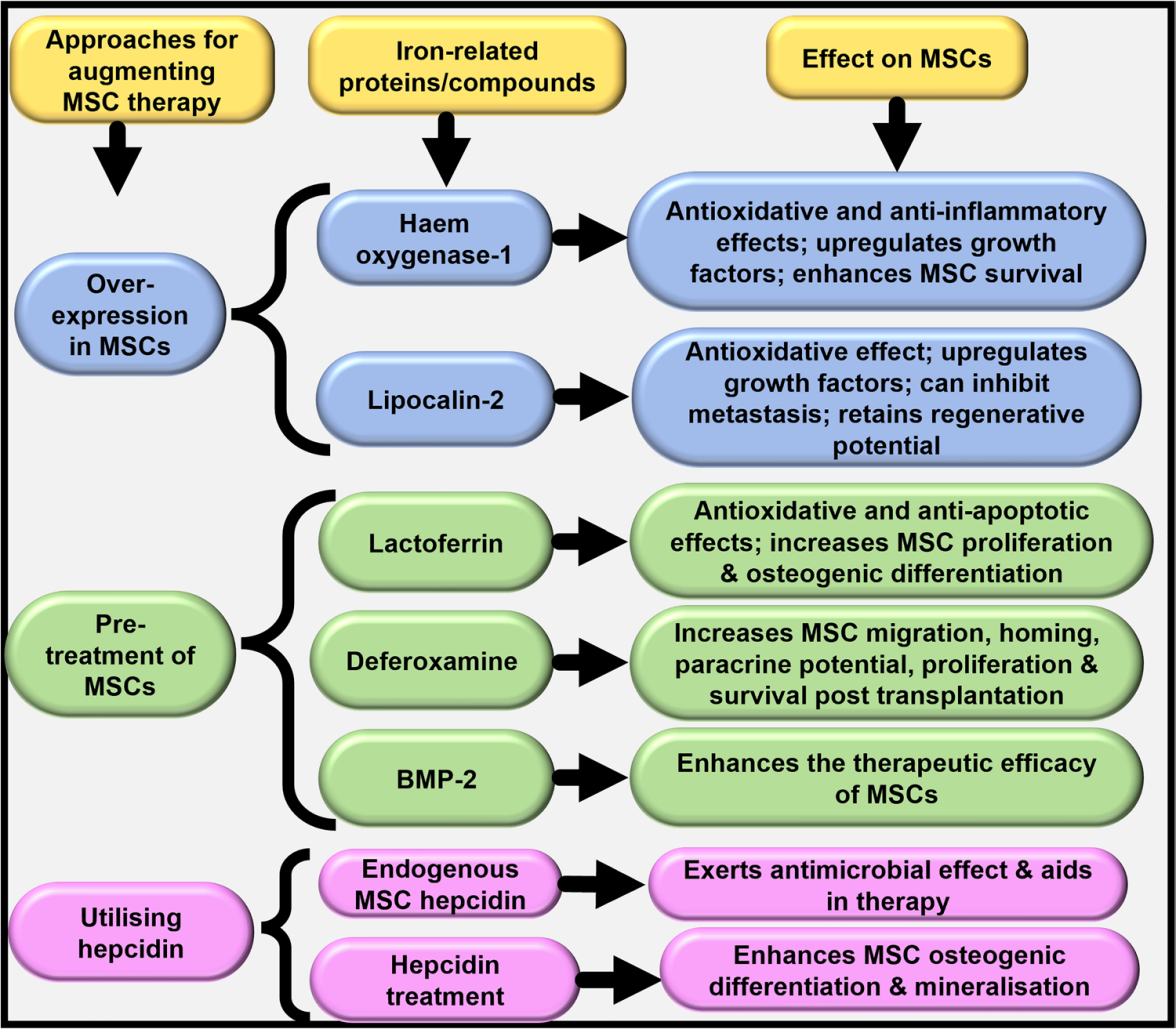
Roles of iron-related proteins and compounds in enhancing MSC therapeutics. The figure presents an overview of the effects of the iron chelator deferoxamine and various iron-related proteins on MSCs and their contributions in enhancing MSC therapeutics

**Fig. 3:**
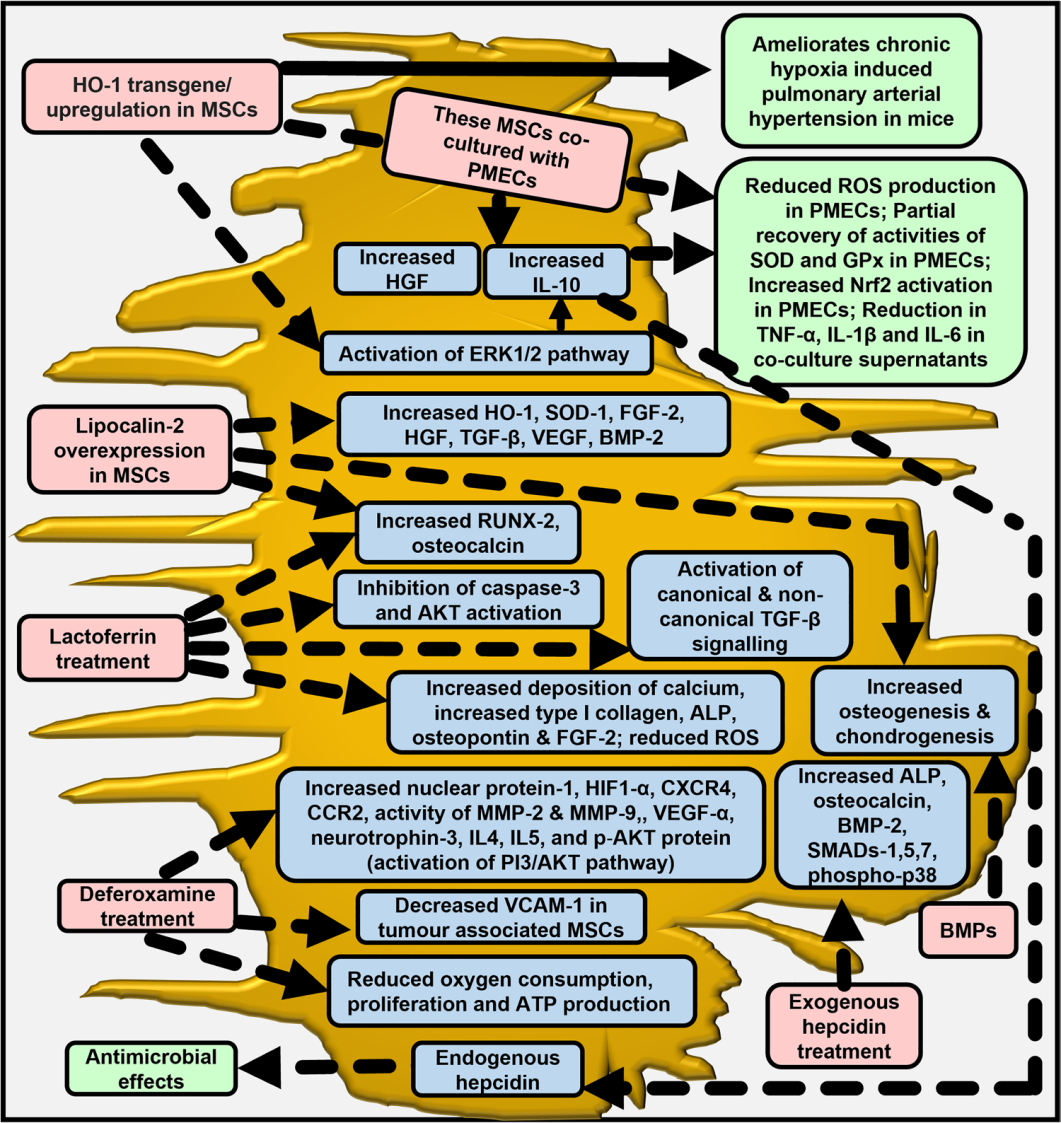
Effects of iron-related proteins and compounds on MSCs. The figure summarises the effects of various iron-related proteins and compounds on MSC biology under unfavourable conditions such as increased ROS production, hypoxia, lipopolysaccharide simulation, iron overload and peroxide-induced stress. ALP: alkaline phosphatase; BMP-2: bone morphogenetic protein-2; CXCR4: C-X-C chemokine receptor type 4; FGF-2: fibroblast growth factor 2; GPx: glutathione peroxidase; HO-1: Haem oxygenase 1; HGF: hepatocyte growth factor; IL: interleukin; MMP: matrix metalloproteinase; Nrf2: nuclear factor erythroid 2–related factor 2; PMECs: pulmonary microvascular endothelial cells; ROS: reactive oxygen species; RUNX-2: Runt-related transcription factor-2; SOD: superoxide dismutase; TGF-β: transforming growth factor beta; TNF-α: tumour necrosis factor alpha; VCAM-1: Vascular cell adhesion protein-1; VEGF: vascular endothelial growth factor

While MSCs are known to execute regenerative and reparative functions, specific iron-related proteins and compounds confer additional beneficial properties upon the MSCs and therefore, their combination with MSCs can greatly augment MSC therapeutics, as summarised in Fig. 2. These beneficial cellular manifestations are at least partly due to the consequent intracellular alterations within the MSCs, as indicated in Fig. 3. These remind us of the IONP-induced MSC alterations [17], and of iron-induced intracellular alterations in hepatic stellate cells [38] and liver carcinoma cells [39], thereby reiterating the capability of iron in altering cellular biology, independent of cell type.

### Haem Oxygenase-1 (HO-1) and its Overexpression in MSCs

HO-1 is inducible by oxidants and inflammatory cytokines, and it is highly expressed in the liver, spleen, and kidneys. It is a rate-limiting enzyme involved in haem metabolism. It degrades cellular haem to produce biliverdin, iron and carbon monoxide. In pathological conditions, the activity of HO-1 can restore homeostasis, and offer cytoprotective effects against oxidative stress through the antioxidant activities of haem-breakdown products biliverdin and bilirubin, and the anti-inflammatory effects of carbon monoxide [40, 41].

Elevation in HO-1 reduced the effects of lipopolysaccharide-induced lung injury in mice [41] and attenuated the post-liver-transplantation-induced acute lung injury in rats [42]. Therefore, Chen et al. hypothesised that due to its antioxidant and anti-inflammatory effects, overexpression of HO-1 in human lungs may reduce injury to pulmonary endothelial cells in acute lung injury [43]. Acute lung injury is characterised by respiratory dysfunction, pulmonary inflammation, edema and damage to endothelium and epithelium. Mortality rate amongst those with acute lung injury and acute respiratory distress syndrome is high. MSC transplantation as a treatment for these syndromes holds promising results [40, 44]. Thus, aiming to help ameliorate the condition, Chen et al. transfected the gene for HO-1 into rat BM-MSCs and observed that while rat BM-MSC co-cultivation with human pulmonary microvascular endothelial cells (PMECs) reduced the lipopolysaccharide-induced damage to PMECs, co-culturing with HO-1-transfected MSCs further reduced this damage. In effect, the release of pro-oxidant and pro-inflammatory cytokines was reduced, production of antioxidant factors was increased and the activation of Nrf2, the key modulator of HO-1 expression was enhanced in PMECs. The rescue effect was partly attributed to the increased production of hepatocyte growth factor (HGF) and IL-10 by the HO-1-transfected rat MSCs [43]. Collectively, this showed that HO-1 overexpression in MSCs could confer additional benefits to MSC transplantation (Fig. 3).

Similar results were observed when BM-MSCs transfected with HO-1 showed protection against the effects of iron-loading (and the consequently generated ROS); again attributed to IL-10 secretion [45]. Also, in-vitro, curcumin-induced pre-induction of HO-1 increased the survival of adipose-derived MSCs, likely via generation of carbon monoxide, and showed protection against hydrogen-peroxide-mediated apoptosis. This promised a feasible strategy to improve MSC therapy [46] as MSC survival post transplantation is often hampered by oxidative stress at the target site. Interestingly, HO-1 transfected BM-MSCs provided protective effects on liver grafts after reduced-size liver transplantation in rats. The effects were mediated through autophagy, as evidenced via upregulation of autophagy-related proteins LC3 and Beclin-1, and increased levels of ERK and p-ERK proteins in the HO-1 transfected BM-MSCs, indicating the activation of the ERK signalling [47] (Fig. 3).

### Lipocalin-2, the Iron Binding Protein, and its Overexpression in MSCs

MSCs show immunomodulatory properties, and their ability to induce immunosuppression and consequently demonstrate therapeutic characteristics in treating inflammatory conditions is known, for e.g., in case of graft-versus-host disease. Here, apoptosis of MSCs has been proposed to be an effector [48, 49]. However, in other cases, the post-transplantation therapeutic benefits of MSCs are dependent on their survival at the site of injury. In certain pre-activation and licensing preparatory protocols, during the pre-transplantation preparatory stages, MSCs are exposed to a nutritionally deficient and hypoxic environment, which leads to elevation in oxidative stress and cytotoxic factors [50]. These factors affect MSC homing and determine treatment efficacy. Lipocalin-2 is an iron binding protein of innate immunity that is produced by various cell types including the MSCs [6, 51]. Usage of lipocalin-2 provides cryoprotection, enables MSCs to resist the environmental challenges in-vivo and thereby augments treatment efficacy (Fig. 2). For example, overexpression of lipocalin-2 in rat BM-MSCs enhanced MSC adhesion and MSC proliferation in-vitro. It also upregulated antioxidants and growth factors and thereby provided protection against stressful microenvironments without affecting their differentiation capacity (Fig. 3). Moreover, lipocalin-2 inhibited the toxic effects of hypoxia, hydrogen peroxide and serum deprivation [52]. Similarly, overexpression of lipocalin-2 in human BM-MSCs decreased peroxide-induced senescence (which otherwise impairs the regenerative potential of MSCs) [53] and thus restored MSC regenerative potential. In another instance, co-culturing BM-MSCs that overexpress lipocalin-2 with kidney-derived cell lines HK-2 and HEK293 prevented cisplatin-induced apoptosis and toxicity in the latter [54]. Levels of antioxidants and growth factors increased in the kidney cells, thereby indicating a reparative function of lipocalin-2 in-vitro. The ability of lipocalin-2 treatment to upregulate BM-MSC osteogenesis (elevate RUNX-2, osteocalcin) and enhance the MSC supportive functions (via elevation of TGF-β, VEGF & BMP-2) [6] (Fig. 3) can be exploited to improve MSC’s therapeutic abilities.

Acute kidney injury does not have an effective treatment till date. MSCs pre-engineered to overexpress lipocalin-2 and then transfused in a rat model of cisplatin-induced acute kidney injury led to enhanced renal function. These cells upregulated several growth factors and markers of proximal tubular epithelium (AQP-1 and CK18), while reducing the markers of kidney injury (KIM-1 and Cystatin C). This indicated new lipocalin-related modalities for acute kidney injury [55]. Moreover, lipocalin-2 (along with prolactin) has been identified as the key BM-derived factor that modulates human MSCs for bone regeneration. Treatment with lipocalin-2 and prolactin postponed cellular senescence of BM-MSCs in-vitro and primed the BM-MSCs for osteogenesis and chondrogenesis. This approach enhanced the repair of skull defects in mice [56]**.** Thus, pre-treatment of MSCs with lipocalin-2 helped maintain their regenerative properties for subsequent applications and the approach demonstrated successful cell-based tissue regeneration in-vivo.

Cancer treatment poses several challenges, one of which is the absence of tumour-specific treatment. While some studies showed that lipocalin-2 promoted carcinogenesis [57–64], other studies demonstrated its anticancer properties [65–69]. To explore its anti-cancer characteristic in combination with the MSC feature of tropism towards tumours [70], MSCs were used as vehicles for targeted delivery of lipocalin-2 for cancer treatment. When BM-MSCs overexpressing lipocalin-2 were transfused in mice with liver metastasis of colon cancer, these cells localised in the metastatic liver and metastasis was inhibited. Vascular endothelial growth factor (VEGF) is the key angiogenic stimulator and angiogenesis is essential for the growth of tumour and its dissemination. BM-MSCs overexpressing lipocalin-2 reduced the expression of VEGF in the metastatic liver [71]. Thus, the lipocalin-2-MSC combination demonstrated the potential for targeted delivery of an anticancer agent for liver metastasis; an approach that can be investigated and applied in treating other cancers.

### Lactoferrin, the Iron Binding Protein in MSC Treatment or in Growth Matrix

Lactoferrin is an antimicrobial and anti-inflammatory iron-binding glycoprotein found in various body secretions [72]. In human MSCs, lactoferrin treatment supressed hydrogen-peroxide-derived ROS levels, senescence, and apoptosis. Lactoferrin exerted this anti-apoptotic effect by inhibiting caspase-3 and AKT activation [73], and emerged as a protector against oxidative stress (Figs. 2 and 3). Also, lactoferrin treatment to human adipose-derived stem cells increased cell proliferation and osteogenic differentiation, as evidenced by increments in calcium deposition and the expressions of alkaline phosphatase (ALP) and RUNX2 (a transcription factor that plays a pivotal role in the differentiation and maturation of osteoblasts) [74]. Thus, lactoferrin treatment to MSCs prior to transplantation can improve the efficacy of MSC therapy for specific diseases.

In bone regeneration applications, the elasticity of the matrix on which the cells are grown in-vitro determine the cellular fate i.e., lineage specification. Bioactive compounds and physical features of the biointerfaces determine MSC osteogenic differentiation. For example, soft gels promote adipogenesis whereas stiff 2-D substrates favour osteogenesis [75]. This is not surprising as the BM-MSCs interact with the extracellular matrix in modulating tissue responses. Utilising a suitable matrix for MSC attachment can reduce the post-transplantation loss of MSCs and greatly enhance MSC therapy for tackling bone injury [75]. This approach was tested in animal models. In a rat model of bone defect, when type II collagen-coated hydroxyapatite/tricalcium phosphate bone scaffolds (substitute) seeded with BM-MSCs were applied to the defect area, these scaffolds showed densely woven bone tissue and marrow formation, demonstrating the significance of the matrix composition in determining bone regeneration capacity [76]. As such, hydroxyapatite is frequently used in biomedical devices for bone-related tissue engineering due to its biocompatible characteristics and its ability to bind to numerous biomolecules without altering their biological functions. Lactoferrin has an anabolic effect on bone and when it was attached to hydroxyapatite nanocrystals, it induced osteogenic differentiation of rabbit BM-MSCs without affecting cell morphology [77]. Similarly, when human MSCs were cultured on surfaces containing lactoferrin and hydroxyapatite (embedded within a biodegradable copolymeric matrix), the osteogenic differentiation of MSCs increased, as evidenced by ALP activity and mineralisation assays [78]. Collectively, this demonstrates the ability of lactoferrin-containing biodegradable composite layers to induce osteogenic differentiation of MSCs and the potential of its usage in bone regeneration applications.

### The Iron Chelator Deferoxamine in MSC Treatment

Deferoxamine is an iron chelator that is often used for scavenging excess iron in iron-overload conditions. It is a hypoxia mimicking agent that can enhance MSC therapeutics (Fig. 2). Pre-transplantation treatment of BM-MSCs with deferoxamine increased cell migration and homing in rat pancreas and thereby increased the efficacy of MSC therapy [79]. This was attributed to the elevations in hypoxia inducible factor-1α (HIF-1α), C-X-C chemokine receptor type 4 (CXCR4) and chemokine receptor 2, and increased activities of matrix metalloproteinases (MMP) 2 and 9 in the deferoxamine-treated BM-MSCs (Fig. 3). Notably, CXCR4 is involved in MSC migration [17] and its expression is often lost during BM-MSC cultivation in-vitro [80]. Thus, deferoxamine-induced elevation of CXCR4 could enhance MSC’s therapeutic potential. Deferoxamine-induced enhancement of migratory and homing potential has also been observed in human adipose-derived MSCs; likely attributed to increments in CXCR4 expression [81]. Similarly, conditioning of rat BM-MSCs with deferoxamine prior to their transplantation in the damaged cochlea of rats improved cell homing via activation of the PI3K/AKT pathway [82]. As such, the downstream subbranches of this pathway that involve mTOR-C1 and FOXO-3 are sensitive to iron [6], which further highlights the significance iron in modulating MSC pathways.

Conditioning human adipose tissue-derived MSCs with deferoxamine increased HIF1-α, and elevated their paracrine potential by increasing neuroprotective factor (nerve growth factor), anti-inflammatory cytokines (IL-4 and IL-5) and pro-angiogenic factor (VEGFα) in the MSC secretome [83]; the latter scenario likely responsible for increased neovascularisation in mice [84]. The usage of adipose-derived MSCs to ameliorate impaired wound healing in diabetics is a promising approach. However, diabetes hampers the reparative functionality of MSCs. Pre-treatment of MSCs derived from the adipose tissue of diabetic patients with deferoxamine increased MSC regenerative potential by elevating HIF1α-induced VEGF production and enhanced the vasculogenic capacity. These effects were replicated in a mice diabetic wound healing model [85]. In addition, deferoxamine exhibited anti-tumour effect in mice tumour-associated MSCs. It inhibited the proliferation of these cells, induced apoptosis and decreased the expression of the adhesion molecule VCAM-1 [86]. VCAM-1 is essential for MSC homing and is known to mediate the interaction between MSC and endothelial cells [87]. However, since Wang et al. observed these effects in mice BM-MSCs as well, the usage of deferoxamine in anti-tumour therapy needs to be addressed with extreme caution, and the bone marrow of patients receiving deferoxamine needs to be thoroughly examined [86].

The differentiation ability, genomic stability, and therapeutic effects of MSCs dependent on culture conditions. Pre-conditioning of MSCs with deferoxamine creates hypoxia-like environment, which promotes MSC proliferation and survival post transplantation, while maintaining cells in an undifferentiated state, and improving therapeutic potential. Mechanistically, deferoxamine inhibits iron-dependent prolyl-4 hydroxylase activity and thereby, HIF1-α hydroxylation and degradation. Accordingly, deferoxamine treatment to BM-MSCs upregulated HIF1-α target genes including VEGF and increased the expression of nuclear protein-1 (Fig. 3). The latter supresses cell cycle via p53 and is involved in cytoprotective autophagy- a lysosomal pathway, which degrades cellular components to maintain cellular homeostasis, while providing substrates for energy metabolism. Thus, deferoxamine-induced elevation in nuclear protein-1 promoted BM-MSC autophagy and thereby autophagy-mediated survival, thus acting as a pro-survival factor [88].

### Bone Morphogenetic Proteins (BMPs), the Modulators of Iron Regulation in MSC Treatment

BMPs are a family of proteins that play an important role during embryogenic development and in adult homeostasis. These regulate important processes such as cell lineage commitment, proliferation, differentiation and apoptosis [89]. Furthermore, BMPs modulate the expression of the iron-hormone hepcidin. In particular, BMP-2 is believed to mediate basal hepcidin induction in the liver via the BMP/SMAD-1/5/8-SMAD4 pathway [90]. BMP-2 has been used in MSC therapy. For instance, rabbits underwent spinal fusion using MSCs with a combination of recombinant human BMP-2 and fibroblast growth factor. Here, they showed high spinal fusion rates with each graft connected to new bone ingrowths. This combination acted as a substitute for autograft in spinal fusion and the results promised more consistent quality of fusion bone than that obtained with an autograft [91]. Thus, BMP-2 enhanced the efficacy of BM-MSCs in mediating spinal fusion. BMPs have been found to induce osteogenesis and chondrogenesis’s in MSCs (Fig. 3) [92]. Usage of BMPs in enhancing MSC therapeutics has been elaborated elsewhere [92].

### Hepcidin, the Iron Hormone and its Utility in MSC Treatment

Hepcidin is an iron hormone produced predominantly by the liver hepatocytes. It is the master regulator of systemic iron homeostasis. Hepcidin is also an antimicrobial peptide [93] and is secreted by the MSCs [94]. This property was exploited to help ameliorate sepsis, a condition that is associated with infection-induced systemic inflammation causing high morbidity and mortality rates. In a mice model of polymicrobial sepsis, the combination of menstrual-derived MSCs and antibiotics enhanced survival, while in-vitro, menstrual-derived MSCs significantly increased hepcidin secretion, thereby reiterating the therapeutic role of hepcidin as an antimicrobial peptide [95]. Thus, MSCs may play an important additional role in tackling infections due to their ability to produce antimicrobial peptides; hepcidin being one of those [94, 96]. As such, the MSCs secrete various cytokines to protect the surrounding tissue from damage by external stimuli [97] and hepcidin secretion by the MSCs could be an part of this function. Interestingly, hepcidin treatment to BM-MSCs can enhance osteogenic differentiation and mineralisation [98] (Fig. 3) and this characteristic can be harnessed when designing MSC therapy for bone loss. Thus, both hepcidin produced endogenously in MSCs and hepcidin added exogenously to MSCs have the potential to enhance MSC therapeutics (Fig. 2).

## Summary

This review collates and examines the roles of iron-related genes and proteins in enhancing MSC detection and therapeutics. Genes of the iron-related proteins ferritin, TfR1 and MagA can be overexpressed in MSCs and used as reporter genes to increase cellular iron accrual and thereby aid in-vivo MSC detection by MRI. Iron-related proteins such as HO-1, lipocalin-2, lactoferrin, BMP-2 and hepcidin, and the iron-related drug deferoxamine exert antioxidant, antiinflammatory, antimicrobial and/or other beneficial effects, thereby potentiating better clinical outcomes when used to enhance MSC therapeutics.

## Data Availability

Not applicable.

## References

[1] Ow Y-LP, Green DR, Hao Z, Mak TW (2008). Cytochrome c: Functions beyond respiration. Nature Reviews Molecular Cell Biology.

[2] Zhao, M., Ma, J., Li, M., Zhang, Y., Jiang, B., Zhao, X., … Qin, S. (2021). Cytochrome P450 Enzymes and Drug Metabolism in Humans. *International Journal of Molecular Sciences*, *22*(23), 12808. 10.3390/ijms222312808 10.3390/ijms222312808 PMC8657965 34884615

[3] Catalase - an overview | ScienceDirect Topics. (n.d.). Retrieved May 9, 2023, from https://www.sciencedirect.com/topics/pharmacology-toxicology-and-pharmaceutical-science/catalase

[4] Cosialls E, El Hage R, Dos Santos L, Gong C, Mehrpour M, Hamaï A (2021). Ferroptosis: Cancer Stem Cells Rely on Iron until “to Die for” It. Cells.

[5] Han, Z., Xu, Z., Chen, L., Ye, D., Yu, Y., Zhang, Y., … Liu, Y. (2020). Iron overload inhibits self‐renewal of human pluripotent stem cells via DNA damage and generation of reactive oxygen species. *FEBS Open Bio*, *10*(5), 726–733. 10.1002/2211-5463.12811 10.1002/2211-5463.12811 PMC7193162 32053740

[6] Mehta KJ (2021). Role of iron and iron-related proteins in mesenchymal stem cells: Cellular and clinical aspects. Journal of Cellular Physiology.

[7] Atluri S, Manchikanti L, Hirsch JA (2020). Expanded Umbilical Cord Mesenchymal Stem Cells (UC-MSCs) as a Therapeutic Strategy in Managing Critically Ill COVID-19 Patients: The Case for Compassionate Use. Pain Physician.

[8] Bai, L., Lennon, D. P., Caplan, A. I., DeChant, A., Hecker, J., Kranso, J., … Miller, R. H. (2012). Hepatocyte growth factor mediates mesenchymal stem cell–induced recovery in multiple sclerosis models. *Nature Neuroscience*, *15*(6), 862–870. 10.1038/nn.3109 10.1038/nn.3109 PMC3427471 22610068

[9] Cancedda R, Giannoni P, Mastrogiacomo M (2007). A tissue engineering approach to bone repair in large animal models and in clinical practice. Biomaterials.

[10] Emadedin, M., Labibzadeh, N., Liastani, M. G., Karimi, A., Jaroughi, N., Bolurieh, T., … Aghdami, N. (2018). Intra-articular implantation of autologous bone marrow-derived mesenchymal stromal cells to treat knee osteoarthritis: a randomized, triple-blind, placebo-controlled phase 1/2 clinical trial. *Cytotherapy*, *20*(10), 1238–1246. 10.1016/j.jcyt.2018.08.005 10.1016/j.jcyt.2018.08.005 30318332

[11] Garza JR, Campbell RE, Tjoumakaris FP, Freedman KB, Miller LS, Santa Maria D, Tucker BS (2020). Clinical Efficacy of Intra-articular Mesenchymal Stromal Cells for the Treatment of Knee Osteoarthritis: A Double-Blinded Prospective Randomized Controlled Clinical Trial. The American Journal of Sports Medicine.

[12] Karussis, D., Karageorgiou, C., Vaknin-Dembinsky, A., Gowda-Kurkalli, B., Gomori, J. M., Kassis, I., … Slavin, S. (2010). Safety and immunological effects of mesenchymal stem cell transplantation in patients with multiple sclerosis and amyotrophic lateral sclerosis. *Archives of Neurology*, *67*(10), 1187–1194. 10.1001/archneurol.2010.248 10.1001/archneurol.2010.248 PMC3036569 20937945

[13] Lee, R. H., Pulin, A. A., Seo, M. J., Kota, D. J., Ylostalo, J., Larson, B. L., … Prockop, D. J. (2009). Intravenous hMSCs improve myocardial infarction in mice because cells embolized in lung are activated to secrete the anti-inflammatory protein TSG-6. *Cell Stem Cell*, *5*(1), 54–63. 10.1016/j.stem.2009.05.003 10.1016/j.stem.2009.05.003 PMC4154377 19570514

[14] Yang, Y., Pang, M., Chen, Y.-Y., Zhang, L.-M., Liu, H., Tan, J., … Rong, L.-M. (2020). Human umbilical cord mesenchymal stem cells to treat spinal cord injury in the early chronic phase: study protocol for a prospective, multicenter, randomized, placebo-controlled, single-blinded clinical trial. *Neural Regeneration Research*, *15*(8), 1532–1538. 10.4103/1673-5374.274347 10.4103/1673-5374.274347 PMC7059580 31997819

[15] Yang, Y.-J., Qian, H.-Y., Huang, J., Geng, Y.-J., Gao, R.-L., Dou, K.-F., … Zhao, S.-H. (2008). Atorvastatin treatment improves survival and effects of implanted mesenchymal stem cells in post-infarct swine hearts. *European Heart Journal*, *29*(12), 1578–1590. 10.1093/eurheartj/ehn167 10.1093/eurheartj/ehn167 18456710

[16] Zhang J, Wu Y, Chen A, Zhao Q (2015). Mesenchymal stem cells promote cardiac muscle repair via enhanced neovascularization. Cellular Physiology and Biochemistry: International Journal of Experimental Cellular Physiology, Biochemistry, and Pharmacology.

[17] Mehta KJ (2022). Iron Oxide Nanoparticles in Mesenchymal Stem Cell Detection and Therapy. Stem Cell Reviews and Reports.

[18] Accomasso L, Gallina C, Turinetto V, Giachino C (2015). Stem Cell Tracking with Nanoparticles for Regenerative Medicine Purposes: An Overview. Stem Cells International.

[19] Harrison RP, Chauhan VM, Onion D, Aylott JW, Sottile V (2019). Intracellular processing of silica-coated superparamagnetic iron nanoparticles in human mesenchymal stem cells. RSC Advances.

[20] Rosenberg JT, Yuan X, Grant S, Ma T (2016). Tracking mesenchymal stem cells using magnetic resonance imaging. Brain Circulation.

[21] Levy, I., Sher, I., Corem-Salkmon, E., Ziv-Polat, O., Meir, A., Treves, A. J., … Rotenstreich, Y. (2015). Bioactive magnetic near Infra-Red fluorescent core-shell iron oxide/human serum albumin nanoparticles for controlled release of growth factors for augmentation of human mesenchymal stem cell growth and differentiation. *Journal of Nanobiotechnology*, *13*, 34. 10.1186/s12951-015-0090-8 10.1186/s12951-015-0090-8 PMC4432958 25947109

[22] Kerans, F. F. A., Lungaro, L., Azfer, A., & Salter, D. M. (2018). The Potential of Intrinsically Magnetic Mesenchymal Stem Cells for Tissue Engineering. *International Journal of Molecular Sciences*, *19*(10). 10.3390/ijms19103159 10.3390/ijms19103159 PMC6214112 30322202

[23] Kircher MF, Gambhir SS, Grimm J (2011). Noninvasive cell-tracking methods. Nature Reviews. Clinical Oncology.

[24] Pereira SM, Herrmann A, Moss D, Poptani H, Williams SR, Murray P, Taylor A (2016). Evaluating the effectiveness of transferrin receptor-1 (TfR1) as a magnetic resonance reporter gene. Contrast Media & Molecular Imaging.

[25] Mu, T., Qin, Y., Liu, B., He, X., Liao, Y., Sun, J., … Cai, J. (2018). In Vitro Neural Differentiation of Bone Marrow Mesenchymal Stem Cells Carrying the FTH1 Reporter Gene and Detection with MRI. *BioMed Research International*, *2018*, 1978602. 10.1155/2018/1978602 10.1155/2018/1978602 PMC6038692 30046590

[26] Kim HS, Woo J, Choi Y, Hwang EH, Choi SK, Cho K-W, Moon WK (2015). Noninvasive MRI and multilineage differentiation capability of ferritin-transduced human mesenchymal stem cells. NMR in Biomedicine.

[27] Huang X, Xue Y, Wu J, Zhan Q, Zhao J (2019). MRI Tracking of SPIO- and Fth1-Labeled Bone Marrow Mesenchymal Stromal Cell Transplantation for Treatment of Stroke. Contrast Media & Molecular Imaging.

[28] Pereira SM, Moss D, Williams SR, Murray P, Taylor A (2015). Overexpression of the MRI Reporter Genes Ferritin and Transferrin Receptor Affect Iron Homeostasis and Produce Limited Contrast in Mesenchymal Stem Cells. International Journal of Molecular Sciences.

[29] Guo, R., Li, Q., Yang, F., Hu, X., Jiao, J., Guo, Y., … Zhang, Y. (2018). In Vivo MR Imaging of Dual MRI Reporter Genes and Deltex-1 Gene-modified Human Mesenchymal Stem Cells in the Treatment of Closed Penile Fracture. *Molecular Imaging and Biology*, *20*(3), 417–427. 10.1007/s11307-017-1128-0 10.1007/s11307-017-1128-0 28971290

[30] Nakamura, C., Burgess, J. G., Sode, K., & Matsunaga, T. (1995). An iron-regulated gene, magA, encoding an iron transport protein of Magnetospirillum sp. strain AMB-1. *The Journal of Biological Chemistry*, *270*(47), 28392–28396. 10.1074/jbc.270.47.28392 10.1074/jbc.270.47.28392 7499342

[31] Uebe R, Henn V, Schüler D (2012). The MagA Protein of Magnetospirilla Is Not Involved in Bacterial Magnetite Biomineralization. Journal of Bacteriology.

[32] Goldhawk, D. E., Lemaire, C., McCreary, C. R., McGirr, R., Dhanvantari, S., Thompson, R. T., … Prato, F. S. (2009). Magnetic resonance imaging of cells overexpressing MagA, an endogenous contrast agent for live cell imaging. *Molecular Imaging*, *8*(3), 129–139. 19723470

[33] Zurkiya O, Chan AWS, Hu X (2008). MagA is sufficient for producing magnetic nanoparticles in mammalian cells, making it an MRI reporter. Magnetic Resonance in Medicine.

[34] Pereira, S. M., Williams, S. R., Murray, P., & Taylor, A. (2016). MS-1 magA: Revisiting Its Efficacy as a Reporter Gene for MRI. *Molecular Imaging*, *15*. 10.1177/1536012116641533 10.1177/1536012116641533 PMC5470133 27118760

[35] Shen Y, Zheng C, Tan Y, Jiang X, Li L (2018). MagA increases MRI sensitivity and attenuates peroxidation-based damage to the bone-marrow haematopoietic microenvironment caused by iron overload. Artificial Cells, Nanomedicine, and Biotechnology.

[36] Son, Y., Cheong, Y.-K., Kim, N.-H., Chung, H.-T., Kang, D. G., & Pae, H.-O. (2011). Mitogen-Activated Protein Kinases and Reactive Oxygen Species: How Can ROS Activate MAPK Pathways? *Journal of Signal Transduction*, *2011*, 792639. 10.1155/2011/792639 10.1155/2011/792639 PMC3100083 21637379

[37] Mehta KJ, Farnaud SJ, Sharp PA (2019). Iron and liver fibrosis: Mechanistic and clinical aspects. World Journal of Gastroenterology.

[38] Mehta KJ, Coombes JD, Briones-Orta M, Manka PP, Williams R, Patel VB, Syn W-K (2018). Iron Enhances Hepatic Fibrogenesis and Activates Transforming Growth Factor-β Signaling in Murine Hepatic Stellate Cells. The American Journal of the Medical Sciences.

[39] Mehta, K. J., & Sharp, P. A. (2020). Iron elevates mesenchymal and metastatic biomarkers in HepG2 cells. *Scientific Reports*, *10*. 10.1038/s41598-020-78348-5 10.1038/s41598-020-78348-5 PMC7736862 33318518

[40] Liang, O. D., Mitsialis, S. A., Chang, M. S., Vergadi, E., Lee, C., Aslam, M., … Kourembanas, S. (2011). Mesenchymal stromal cells expressing heme oxygenase-1 reverse pulmonary hypertension. *Stem Cells (Dayton, Ohio)*, *29*(1), 99–107. 10.1002/stem.548 10.1002/stem.548 PMC3422740 20957739

[41] Yin, H., Li, X., Gong, Q., Jin, X., Gu, H., Yuan, B., … Zhu, J. (2010). Heme oxygenase-1 upregulation improves lipopolysaccharide-induced acute lung injury involving suppression of macrophage migration inhibitory factor. *Molecular Immunology*, *47*(15), 2443–2449. 10.1016/j.molimm.2010.06.013 10.1016/j.molimm.2010.06.013 20638132

[42] Chi, X., Guo, N., Yao, W., Jin, Y., Gao, W., Cai, J., & Hei, Z. (2016). Induction of heme oxygenase-1 by hemin protects lung against orthotopic autologous liver transplantation-induced acute lung injury in rats. *Journal of Translational Medicine*, *14*. 10.1186/s12967-016-0793-0 10.1186/s12967-016-0793-0 PMC4736160 26838179

[43] Chen X, Zhang Y, Wang W, Liu Z, Meng J, Han Z (2018). Mesenchymal Stem Cells Modified with Heme Oxygenase-1 Have Enhanced Paracrine Function and Attenuate Lipopolysaccharide-Induced Inflammatory and Oxidative Damage in Pulmonary Microvascular Endothelial Cells. Cellular Physiology and Biochemistry: International Journal of Experimental Cellular Physiology, Biochemistry, and Pharmacology.

[44] Matthay MA (2015). Therapeutic Potential of Mesenchymal Stromal Cells for Acute Respiratory Distress Syndrome. Annals of the American Thoracic Society.

[45] Yu, Z. Y., Ma, D., He, Z. C., Liu, P., Huang, J., Fang, Q., … Wang, J. S. (2018). Heme oxygenase-1 protects bone marrow mesenchymal stem cells from iron overload through decreasing reactive oxygen species and promoting IL-10 generation. *Experimental Cell Research*, *362*(1), 28–42. 10.1016/j.yexcr.2017.10.029 10.1016/j.yexcr.2017.10.029 29111167

[46] Cremers, N. A. J., Lundvig, D. M. S., van Dalen, S. C. M., Schelbergen, R. F., van Lent, P. L. E. M., Szarek, W. A., … Wagener, F. A. D. T. G. (2014). Curcumin-induced heme oxygenase-1 expression prevents H2O2-induced cell death in wild type and heme oxygenase-2 knockout adipose-derived mesenchymal stem cells. *International Journal of Molecular Sciences*, *15*(10), 17974–17999. 10.3390/ijms151017974 10.3390/ijms151017974 PMC4227200 25299695

[47] Wang, R., Shen, Z., Yang, L., Yin, M., Zheng, W., Wu, B., … Song, H. (2017). Protective effects of heme oxygenase-1-transduced bone marrow-derived mesenchymal stem cells on reduced‑size liver transplantation: Role of autophagy regulated by the ERK/mTOR signaling pathway. *International Journal of Molecular Medicine*, *40*(5), 1537–1548. 10.3892/ijmm.2017.3121 10.3892/ijmm.2017.3121 PMC5627878 28901391

[48] Cheung TS, Galleu A, von Bonin M, Bornhäuser M, Dazzi F (2019). Apoptotic mesenchymal stromal cells induce prostaglandin E2 in monocytes: Implications for the monitoring of mesenchymal stromal cell activity. Haematologica.

[49] Müller, L., Tunger, A., Wobus, M., von Bonin, M., Towers, R., Bornhäuser, M., … Schmitz, M. (2021). Immunomodulatory Properties of Mesenchymal Stromal Cells: An Update. *Frontiers in Cell and Developmental Biology*, *9*, 637725. 10.3389/fcell.2021.637725 10.3389/fcell.2021.637725 PMC7900158 33634139

[50] Zhu W, Chen J, Cong X, Hu S, Chen X (2006). Hypoxia and serum deprivation-induced apoptosis in mesenchymal stem cells. Stem Cells (Dayton, Ohio).

[51] Gupta N, Krasnodembskaya A, Kapetanaki M, Mouded M, Tan X, Serikov V, Matthay MA (2012). Mesenchymal stem cells enhance survival and bacterial clearance in murine Escherichia coli pneumonia. Thorax.

[52] Halabian R, Tehrani HA, Jahanian-Najafabadi A, Habibi Roudkenar M (2013). Lipocalin-2-mediated upregulation of various antioxidants and growth factors protects bone marrow-derived mesenchymal stem cells against unfavorable microenvironments. Cell Stress & Chaperones.

[53] Bahmani B, Roudkenar MH, Halabian R, Jahanian-Najafabadi A, Amiri F, Jalili MA (2014). Lipocalin 2 decreases senescence of bone marrow-derived mesenchymal stem cells under sub-lethal doses of oxidative stress. Cell Stress & Chaperones.

[54] Halabian R, Roudkenar MH, Jahanian-Najafabadi A, Hosseini KM, Tehrani HA (2015). Co-culture of bone marrow-derived mesenchymal stem cells overexpressing lipocalin 2 with HK-2 and HEK293 cells protects the kidney cells against cisplatin-induced injury. Cell Biology International.

[55] Roudkenar, M. H., Halabian, R., Tehrani, H. A., Amiri, F., Jahanian-Najafabadi, A., Roushandeh, A. M., … kuwahara, Y. (2018). Lipocalin 2 enhances mesenchymal stem cell-based cell therapy in acute kidney injury rat model. *Cytotechnology*, *70*(1), 103–117. 10.1007/s10616-017-0107-2 10.1007/s10616-017-0107-2 PMC5809639 28573544

[56] Tsai T-L, Li W-J (2017). Identification of Bone Marrow-Derived Soluble Factors Regulating Human Mesenchymal Stem Cells for Bone Regeneration. Stem Cell Reports.

[57] Gomez-Chou, S. B., Swidnicka-Siergiejko, A. K., Badi, N., Chavez-Tomar, M., Lesinski, G. B., Bekaii-Saab, T., … Cruz-Monserrate, Z. (2017). Lipocalin-2 Promotes Pancreatic Ductal Adenocarcinoma by Regulating Inflammation in the Tumor Microenvironment. *Cancer Research*, *77*(10), 2647–2660. 10.1158/0008-5472.CAN-16-1986 10.1158/0008-5472.CAN-16-1986 PMC5441230 28249896

[58] Hu C, Yang K, Li M, Huang W, Zhang F, Wang H (2018). Lipocalin 2: A potential therapeutic target for breast cancer metastasis. OncoTargets and therapy.

[59] Mannelqvist, M., Stefansson, I. M., Wik, E., Kusonmano, K., Raeder, M. B., Øyan, A. M., … Akslen, L. A. (2012). Lipocalin 2 expression is associated with aggressive features of endometrial cancer. *BMC Cancer*, *12*, 169. 10.1186/1471-2407-12-169 10.1186/1471-2407-12-169 PMC3493289 22559235

[60] Mongre, R. K., Sodhi, S. S., Sharma, N., Ghosh, M., Kim, J. H., Kim, N., … Jeong, D. K. (2015). Epigenetic induction of epithelial to mesenchymal transition by LCN2 mediates metastasis and tumorigenesis, which is abrogated by NF-κB inhibitor BRM270 in a xenograft model of lung adenocarcinoma. *International Journal of Oncology*, *48*(1), 84–98. 10.3892/ijo.2015.3245 10.3892/ijo.2015.3245 PMC4734607 26573874

[61] Rodvold JJ, Mahadevan NR, Zanetti M (2012). Lipocalin 2 in cancer: When good immunity goes bad. Cancer Letters.

[62] Shi H, Gu Y, Yang J, Xu L, Mi W, Yu W (2008). Lipocalin 2 promotes lung metastasis of murine breast cancer cells. Journal of Experimental & Clinical Cancer Research : CR.

[63] Yang J, Moses MA (2009). Lipocalin 2: A Multifaceted Modulator of Human Cancer. Cell Cycle.

[64] Yang, J., Bielenberg, D. R., Rodig, S. J., Doiron, R., Clifton, M. C., Kung, A. L., … Moses, M. A. (2009). Lipocalin 2 promotes breast cancer progression. *Proceedings of the National Academy of Sciences of the United States of America*, *106*(10), 3913–3918. 10.1073/pnas.0810617106 10.1073/pnas.0810617106 PMC2656179 19237579

[65] Feng, M., Feng, J., Chen, W., Wang, W., Wu, X., Zhang, J., … Lai, M. (2016). Lipocalin2 suppresses metastasis of colorectal cancer by attenuating NF-κB-dependent activation of snail and epithelial mesenchymal transition. *Molecular Cancer*, *15*(1), 77. 10.1186/s12943-016-0564-9 10.1186/s12943-016-0564-9 PMC5135816 27912767

[66] Lee H-J, Lee E-K, Lee K-J, Hong S-W, Yoon Y, Kim J-S (2006). Ectopic expression of neutrophil gelatinase-associated lipocalin suppresses the invasion and liver metastasis of colon cancer cells. International Journal of Cancer.

[67] Lin, C.-W., Yang, W.-E., Lee, W.-J., Hua, K.-T., Hsieh, F.-K., Hsiao, M., … Chien, M.-H. (2016). Lipocalin 2 prevents oral cancer metastasis through carbonic anhydrase IX inhibition and is associated with favourable prognosis. *Carcinogenesis*, *37*(7), 712–722. 10.1093/carcin/bgw050 10.1093/carcin/bgw050 27207653

[68] Moschen, A. R., Gerner, R. R., Wang, J., Klepsch, V., Adolph, T. E., Reider, S. J., … Tilg, H. (2016). Lipocalin 2 Protects from Inflammation and Tumorigenesis Associated with Gut Microbiota Alterations. *Cell Host & Microbe*, *19*(4), 455–469. 10.1016/j.chom.2016.03.007 10.1016/j.chom.2016.03.007 27078067

[69] Tong, Z., Kunnumakkara, A. B., Wang, H., Matsuo, Y., Diagaradjane, P., Harikumar, K. B., … Guha, S. (2008). Neutrophil gelatinase-associated lipocalin: a novel suppressor of invasion and angiogenesis in pancreatic cancer. *Cancer Research*, *68*(15), 6100–6108. 10.1158/0008-5472.CAN-08-0540 10.1158/0008-5472.CAN-08-0540 PMC2714276 18676832

[70] Chen Y, He Y, Wang X, Lu F, Gao J (2019). Adipose-derived mesenchymal stem cells exhibit tumor tropism and promote tumorsphere formation of breast cancer cells. Oncology Reports.

[71] Harati, M. D., Amiri, F., Jaleh, F., Mehdipour, A., Harati, M. D., Molaee, S., … Roudkenar, M. H. (2015). Targeting delivery of lipocalin 2-engineered mesenchymal stem cells to colon cancer in order to inhibit liver metastasis in nude mice. *Tumour Biology: The Journal of the International Society for Oncodevelopmental Biology and Medicine*, *36*(8), 6011–6018. 10.1007/s13277-015-3277-6 10.1007/s13277-015-3277-6 25740061

[72] Cornish, J., Callon, K. E., Naot, D., Palmano, K. P., Banovic, T., Bava, U., … Reid, I. R. (2004). Lactoferrin is a potent regulator of bone cell activity and increases bone formation in vivo. *Endocrinology*, *145*(9), 4366–4374. 10.1210/en.2003-1307 10.1210/en.2003-1307 15166119

[73] Park, S. Y., Jeong, A.-J., Kim, G.-Y., Jo, A., Lee, J. E., Leem, S.-H., … Chung, J. W. (2017). Lactoferrin Protects Human Mesenchymal Stem Cells from Oxidative Stress-Induced Senescence and Apoptosis. *Journal of Microbiology and Biotechnology*, *27*(10), 1877–1884. 10.4014/jmb.1707.07040 10.4014/jmb.1707.07040 28870012

[74] Ying, X., Cheng, S., Wang, W., Lin, Z., Chen, Q., Zhang, W., … Zhu Lu, C. (2012). Effect of lactoferrin on osteogenic differentiation of human adipose stem cells. *International Orthopaedics*, *36*(3), 647–653. 10.1007/s00264-011-1303-x 10.1007/s00264-011-1303-x PMC3291782 21713451

[75] Pittenger, M. F., Discher, D. E., Péault, B. M., Phinney, D. G., Hare, J. M., & Caplan, A. I. (2019). Mesenchymal stem cell perspective: cell biology to clinical progress. *Npj Regenerative Medicine*, *4*(1), 1–15. 10.1038/s41536-019-0083-6 10.1038/s41536-019-0083-6 PMC6889290 31815001

[76] Chiu L-H, Lai W-FT, Chang S-F, Wong C-C, Fan C-Y, Fang C-L, Tsai Y-H (2014). The effect of type II collagen on MSC osteogenic differentiation and bone defect repair. Biomaterials.

[77] Montesi M, Panseri S, Iafisco M, Adamiano A, Tampieri A (2015). Effect of hydroxyapatite nanocrystals functionalized with lactoferrin in osteogenic differentiation of mesenchymal stem cells. Journal of Biomedical Materials Research Part A.

[78] Icriverzi, M., Bonciu, A., Rusen, L., Sima, L. E., Brajnicov, S., Cimpean, A., … Roseanu, A. (2019). Human Mesenchymal Stem Cell Response to Lactoferrin-based Composite Coatings. *Materials*, *12*(20). 10.3390/ma12203414 10.3390/ma12203414 PMC6829495 31635291

[79] Najafi R, Sharifi AM (2013). Deferoxamine preconditioning potentiates mesenchymal stem cell homing in vitro and in streptozotocin-diabetic rats. Expert Opinion on Biological Therapy.

[80] Musiał-Wysocka A, Kot M, Majka M (2019). The Pros and Cons of Mesenchymal Stem Cell-Based Therapies. Cell Transplantation.

[81] Heirani-Tabasi, A., Naderi-Meshkin, H., Matin, M. M., Mirahmadi, M., Shahriyari, M., Ahmadiankia, N., … Bahrami, A. R. (2018). Augmented migration of mesenchymal stem cells correlates with the subsidiary CXCR4 variant. *Cell Adhesion & Migration*, *12*(2), 118–126. 10.1080/19336918.2016.1243643 10.1080/19336918.2016.1243643 PMC5927652 29466916

[82] Peyvandi, A. A., Abbaszadeh, H.-A., Roozbahany, N. A., Pourbakht, A., Khoshsirat, S., Niri, H. H., … Niknazar, S. (2018). Deferoxamine promotes mesenchymal stem cell homing in noise-induced injured cochlea through PI3K/AKT pathway. *Cell Proliferation*, *51*(2), e12434. 10.1111/cpr.12434 10.1111/cpr.12434 PMC6528934 29341316

[83] Oses, C., Olivares, B., Ezquer, M., Acosta, C., Bosch, P., Donoso, M., … Ezquer, F. (2017). Preconditioning of adipose tissue-derived mesenchymal stem cells with deferoxamine increases the production of pro-angiogenic, neuroprotective and anti-inflammatory factors: Potential application in the treatment of diabetic neuropathy. *PLoS ONE*, *12*(5). 10.1371/journal.pone.0178011 10.1371/journal.pone.0178011 PMC5438173 28542352

[84] Wahl EA, Schenck TL, Machens H-G, Balmayor ER (2016). VEGF released by deferoxamine preconditioned mesenchymal stem cells seeded on collagen-GAG substrates enhances neovascularization. Scientific Reports.

[85] Hopfner, U., Maan, Z. N., Hu, M. S., Aitzetmüller, M. M., Zaussinger, M., Kirsch, M., … Duscher, D. (2020). Deferoxamine enhances the regenerative potential of diabetic Adipose Derived Stem Cells. *Journal of Plastic, Reconstructive & Aesthetic Surgery*. 10.1016/j.bjps.2020.02.045 10.1016/j.bjps.2020.02.045 32418841

[86] Wang G, Shen G, Yin T (2017). In vitro assessment of deferoxamine on mesenchymal stromal cells from tumor and bone marrow. Environmental Toxicology and Pharmacology.

[87] Jung EM, Kwon O, Kwon K-S, Cho YS, Rhee SK, Min J-K, Oh D-B (2011). Evidences for correlation between the reduced VCAM-1 expression and hyaluronan synthesis during cellular senescence of human mesenchymal stem cells. Biochemical and Biophysical Research Communications.

[88] Matsunaga, K., Fujisawa, K., Takami, T., Burganova, G., Sasai, N., Matsumoto, T., … Sakaida, I. (2019). NUPR1 acts as a pro-survival factor in human bone marrow-derived mesenchymal stem cells and is induced by the hypoxia mimetic reagent deferoxamine. *Journal of Clinical Biochemistry and Nutrition*, *64*(3), 209–216. 10.3164/jcbn.18-112 10.3164/jcbn.18-112 PMC6529697 31138954

[89] Katagiri, T., & Watabe, T. (2016). Bone Morphogenetic Proteins. *Cold Spring Harbor Perspectives in Biology*, *8*(6). 10.1101/cshperspect.a021899 10.1101/cshperspect.a021899 PMC4888821 27252362

[90] Canali S, Wang C-Y, Zumbrennen-Bullough KB, Bayer A, Babitt JL (2017). Bone morphogenetic protein 2 controls iron homeostasis in mice independent of Bmp6. American Journal of Hematology.

[91] Minamide A, Yoshida M, Kawakami M, Okada M, Enyo Y, Hashizume H, Boden SD (2007). The effects of bone morphogenetic protein and basic fibroblast growth factor on cultured mesenchymal stem cells for spine fusion. Spine.

[92] Scarfì S (2016). Use of bone morphogenetic proteins in mesenchymal stem cell stimulation of cartilage and bone repair. World Journal of Stem Cells.

[93] Ganz T (2011). Hepcidin and iron regulation, 10 years later. Blood.

[94] Esfandiyari, R., Halabian, R., Behzadi, E., Sedighian, H., Jafari, R., & Imani Fooladi, A. A. (2019). Performance evaluation of antimicrobial peptide ll-37 and hepcidin and β-defensin-2 secreted by mesenchymal stem cells. *Heliyon*, *5*(10), e02652. 10.1016/j.heliyon.2019.e02652 10.1016/j.heliyon.2019.e02652 PMC6820248 31687504

[95] Alcayaga-Miranda F, Cuenca J, Martin A, Contreras L, Figueroa FE, Khoury M (2015). Combination therapy of menstrual derived mesenchymal stem cells and antibiotics ameliorates survival in sepsis. Stem Cell Research & Therapy.

[96] Chow L, Johnson V, Impastato R, Coy J, Strumpf A, Dow S (2020). Antibacterial activity of human mesenchymal stem cells mediated directly by constitutively secreted factors and indirectly by activation of innate immune effector cells. Stem Cells Translational Medicine.

[97] Kyurkchiev D, Bochev I, Ivanova-Todorova E, Mourdjeva M, Oreshkova T, Belemezova K, Kyurkchiev S (2014). Secretion of immunoregulatory cytokines by mesenchymal stem cells. World Journal of Stem Cells.

[98] Lu H, Lian L, Shi D, Zhao H, Dai Y (2015). Hepcidin promotes osteogenic differentiation through the bone morphogenetic protein 2/small mothers against decapentaplegic and mitogen-activated protein kinase/P38 signaling pathways in mesenchymal stem cells. Molecular Medicine Reports.

